# Inhibition of mannan-binding lectin associated serine protease (MASP)-2 reduces the cognitive deficits in a mouse model of severe traumatic brain injury

**DOI:** 10.1186/s12974-024-03133-4

**Published:** 2024-05-28

**Authors:** Domenico Mercurio, Francesca Pischiutta, Serena Seminara, Francesca Tribuzio, Ilaria Lisi, Laura Pasetto, Valentina Bonetto, Maria-Grazia De Simoni, Wilhelm Schwaeble, Sadam Yaseen, Thomas Dudler, Elisa R. Zanier, Stefano Fumagalli

**Affiliations:** 1 Department of Acute Brain and Cardiovascular Injury, Istituto di Ricerche Farmacologiche Mario Negri IRCCS, Milan, Italy; 2 Department of Neuroscience, Istituto di Ricerche Farmacologiche Mario Negri IRCCS, Milan, Italy; 3 Department of Veterinary Medicine, School of Biological Sciences, University of Cambridge, Cambridge, UK; 4 Omeros Corporation, Seattle, WA USA

**Keywords:** Complement system, Traumatic brain injury, Pharmacology, Mannan-binding lectin-associated serine proteases

## Abstract

The lectin pathway (LP) of complement mediates inflammatory processes linked to tissue damage and loss of function following traumatic brain injury (TBI). LP activation triggers a cascade of proteolytic events initiated by LP specific enzymes called MASPs (for Mannan-binding lectin Associated Serine Proteases). Elevated serum and brain levels of MASP-2, the effector enzyme of the LP, were previously reported to be associated with the severity of tissue injury and poor outcomes in patients with TBI. To evaluate the therapeutic potential of LP inhibition in TBI, we first conducted a pilot study testing the effect of an inhibitory MASP-2 antibody (α-MASP-2), administered systemically at 4 and 24 h post-TBI in a mouse model of controlled cortical impact (CCI). Treatment with α-MASP-2 reduced sensorimotor and cognitive deficits for up to 5 weeks post-TBI. As previous studies by others postulated a critical role of MASP-1 in LP activation, we conducted an additional study that also assessed treatment with an inhibitory MASP-1 antibody (α-MASP-1). A total of 78 mice were treated intraperitoneally with either α-MASP-2, or α-MASP-1, or an isotype control antibody 4 h and 24 h after TBI or sham injury. An amelioration of the cognitive deficits assessed by Barnes Maze, prespecified as the primary study endpoint, was exclusively observed in the α-MASP-2-treated group. The behavioral data were paralleled by a reduction of the lesion size when evaluated histologically and by reduced systemic LP activity. Our data suggest that inhibition of the LP effector enzyme MASP-2 is a promising treatment strategy to limit neurological deficits and tissue loss following TBI. Our work has translational value because a MASP-2 antibody has already completed multiple late-stage clinical trials in other indications and we used a clinically relevant treatment protocol testing the therapeutic mechanism of MASP-2 inhibition in TBI.

## Background

Traumatic brain injury (TBI) is a leading cause of death and disability worldwide. The most recent Global Burden of Disease reported an annual incidence across Europe of at least 4 million [1]. TBI represents a high burden to patients, society, and health-care systems, but there are still limited treatment options available.

TBI pathophysiology is characterized by secondary events that follow the initial impact and evolve over time and across brain areas. The initial response to TBI includes protective endogenous mechanisms as well as detrimental cascades, with the balance contributing to neurological worsening or recovery [2]. Post-TBI inflammation may contribute to secondary pathophysiological mechanisms in opposite manners, because it helps limit the spread of toxic signals and supports tissue remodeling, but can also aggravate TBI consequences by releasing pro-inflammatory mediators.

Activation of the complement system triggers an inflammatory cascade that contributes to the exacerbation of TBI damage, leading to tissue damage and loss of neurological functions [3, 4]. Depending on the initiating event, complement activation may occur through three different pathways: the classical pathway (CP), the alternative pathway (AP) and the lectin pathway (LP), each composed of specific initiators and effector enzymes. Non-self-carbohydrate structures or acetylated proteins exposed on the surface of damaged cells, including apoptotic or necrotic cells and stressed endothelium [5], represent molecular patterns recognized by LP recognition subcomponents. Binding of LP recognition subcomponents such as the collectins mannan-binding lectin (MBL) and collectin-10 (CL-L1) and collectin-11 (CL-K1), or ficolins, such as ficolin-1 (M-ficolin), ficolin-2 (L-ficolin) or ficolin-3 (H-ficolin) and MBL-A and MBL-C, collectin-10 and collectin-11 and Ficolin A and Ficolin C in rodents leads to activation of MASPs and initiates complement activation via the LP.

Among MASPs, it was established that only MASP-2 can cleave both C2 and C4 to generate a C4b2a type C3 convertase, while MASP-1 cleaves C2, but is unable to cleave C4 and MASP-3 cannot cleave either C2 or C4. MASP-2 can therefore be considered as the single key initiator of the LP as it can autoactivate and generate a C3 convertase by its own, with MASP-1 acting as an auxiliary enzyme that provides some of the activated C2 component for the convertase [6, 7]. As such, MASP-2 and MASP-1 are both potential targets to dampen LP activation.

Previous studies pointed to activation of the LP as a critical contributor to TBI pathophysiology [8–10]. LP recognition molecules are found decorating the vessels pertinent to the injured areas in models of acute brain injury such as stroke [11] and TBI [9, 10]. In specimens obtained from patients with severe TBI undergoing the surgical removal of the lesion, the LP recognition molecules MBL and ficolin-1, -2, and −3 were all found to be associated with the affected blood vessels [10]. Importantly, MASP-2 was present at higher levels on TBI vessels when compared to brain specimens obtained from controls, including gliomas, or autoptic brains of people who died due to extracranial causes [8]. Clinical observations by Osthoff et al. reported that elevated blood levels of MASP-2 early after TBI of all severities were associated with poor 90-day outcomes [12].

In our previous work, we modeled TBI by controlled cortical impact (CCI) in mice carrying gene deletions of key LP components to study the effects on the neurological functions and pathology. Using MASP-2 (Masp2^−/−^), ficolin-A (Fcna^−/−^), CL-11 (Colec11^−/−^), MASP-1/3 (Masp1^−/−^), MBL-C (Mbl2^−/−^), MBL-A (Mbl1^−/−^) or MBL (Mbl1^−/−^ / Mbl2^−/−^)-deficient male C57BL/6J or WT mice, we reported that the absence of the essential LP effector enzyme MASP-2 was associated with the highest degree of protection after TBI [13].

Mounting evidence suggests that MASP-2 represents a superior therapeutic target for ameliorating TBI consequences. Since MASP-2 is a low abundance plasma protein produced exclusively by the liver, systemic inhibition of the LP using anti-MASP-2 antibody (α-MASP-2) which blocks MASP-2 functional activity is eminently feasible. To further evaluate the therapeutic potential of MASP-2 inhibition for the treatment of TBI, we tested therapeutic intervention protocols using a recombinant MASP-2 antibody that was previously shown to ameliorate ischemic stroke deficits [14] and COVID-19-induced brain inflammation [15] in mouse models, and compared it to an anti-MASP-1 antibody. Our data presented herein suggest that acute α-MASP-2 treatment is a promising new therapeutic approach to improve recovery following TBI.

## Methods

### Animals

We used male C57BL/6J mice (Charles River, Italy), 9 weeks of age, weighing 21–26 g for the experiments. Procedures involving animals and their care were conducted in conformity with institutional guidelines, which comply to National (D.L. n.116, G.U. suppl. 40, 18 February 1992) and international laws and policies (EEC Council Directive 86/609, OJ L 358,1; Dec.12,1987; NIH Guide for the Care and Use of Laboratory Animals, U.S. National Research Council 1996). The project was approved by the Italian Ministry of Health with the codes No. 9F5F5.148 (approval number 80/2020-PR) for the first part of the study and No. 9F5F5.206 (approval number 117/2022-PR) for the second part of the study.

Mice were housed 5 per cage and kept at a constant temperature (21° ± 1 °C) and relative humidity (60%) with a regular light/dark schedule (7am-7pm). Food (Altromin pellets for mice) and water were available ad libitum.

### Experimental TBI

Mice were anesthetized with isoflurane inhalation (induction 5%; maintenance 2%) in an N_2_O/O_2_ (70%/30%) mixture and placed in a stereotaxic frame. Rectal temperature was maintained at 37 °C using a feedback-controlled heating pad. Mice were subjected to craniectomy followed by CCI brain injury as previously described [16]. Briefly, the injury was induced using a 3-mm diameter rigid impactor driven by an electromagnetic piston (Leica, Impact One) mounted at an angle of 20° from the vertical plane and applied vertically to the exposed dura mater, between bregma and lambda, over the left parieto-temporal cortex (antero-posteriority: −2.5 mm, laterality: −2.5 mm), at impactor velocity of 5 m/s and deformation depth 2 mm, resulting in a severe level of injury [9, 17]. The craniotomy was covered with a cranioplasty and the scalp sutured. Sham-operated mice received identical anesthesia and surgery without brain injury.

HG-4, a derivative of the clinically used MASP-2 inhibitor narsoplimab, was used for MASP-2 inhibition in these mouse studies while antibodies 86C3 and motavizumab were used for MASP-1 inhibition and isotype control treatment, respectively [14, 18]. Antibody treatments were administered by intraperitoneal (i.p.) injection at a dose of 10 mg/kg of body weight at 4- and 24-hours post TBI.

### Behavioral tests

#### Sensorimotor deficits

*Neuroscore*: mice were scored from 4 (normal) to 0 (severely impaired) for each of the following: forelimb function, hindlimb function during walking on a grid, and resistance to lateral left pulse. The best possible score, corresponding to no deficits, was 12 [13].

*Simple Neuroassessment of Asymmetric imPairment (SNAP)*: Mice were evaluated using eight tests measuring: interaction with the handler, grip strength, visual placing, pacing/circling, gait and posture, head tilt, visual field, and coordination and proprioception. Scores ranged from 0 (normal) to 5 (severely impaired) for each test and were summed to give an overall score that ranged from 0 (best) to 40 (worst) [19].

### Cognitive deficits

*Barnes maze*: the test assessed spatial learning and memory. The maze consists of a circular platform with multiple holes around its perimeter, only one of which leads to an escape tunnel or a safe area. The test started with a habituation trial (day 1) during which the mouse was placed in the center of the empty arena under a beaker for 30 s, then guided to the escape tunnel over 10–15 s by slowly moving the beaker and allowing 2 min to enter the escape tunnel spontaneously. The mouse was allowed to stay in the escape for 1 min and then was taken back to its home cage. In the learning phase (days 2–4), mice were placed for 10 s in the center of the arena and then allowed to explore the arena for at least 2 min to find the escape (primary latency) and to enter it (secondary latency). The test phase (day 5) consisted of the removal of the escape tunnel from the maze to assess the primary latency [20]. The escape was sealed (‘false escape’) to prevent the mouse from falling through the hole. The ability of the mouse to find the escape within the 2-minute time was recorded.

### Anxiety-like behavior

*Elevated plus maze (EPM)*: the test (set up in-house) measured disinhibition and anxiety-like behaviors. The test consisted of two open and two closed arms (each 35 cm × 5.5 cm) and a central platform (5.5 cm × 5.5 cm) elevated 60 cm above the ground. Mice were acclimatized in the room for 1 h before testing then placed on the central platform facing an open arm, and their movements were recorded for 5 min. Video recording and time spent in the closed and open arms were measured by Ethovision XT, 14 (Noldus Information Technology, Wageningen, The Netherlands).

### Health score

To obtain a comparative analysis of the functional outcome, each experimental subject was rated according to a health score calculated on functional outcomes, as shown previously [13]. Briefly, the Barnes maze, EPM, neuroscore, and SNAP performance data sets obtained in motavizumab-treated mice (*N* = 19) were stratified into four groups according to quartiles. Each quartile was attributed a score ranging from 4 (best) to 1 (worst outcome). Each mouse obtained a final score, which was the sum of the weighted scores of the four parameters, each accounting for 25% of the final score. The effect size (odds ratio) was calculated by a Chi square test using the Woolf logit interval for computing the 95% confidence interval (CI), stratifying mice in terms of good outcome (> 3) versus bad outcome (≤ 3). Odds ratios with 95% CI were reported in the Forest plot to visualize the strength of the association between the treatments and the functional outcome.

### Sample collection

Blood for longitudinal assessment was collected from the submandibular vein at 4 days and 6 weeks after TBI. Clotting and complement activation was prevented by collecting samples into BD Microtainer™ Tubes containing K_2_EDTA as anticoagulant (BD, ref 365,975). Blood was centrifuged at 2000 × g for 15 min at 4 °C and plasma was then stored at − 80 °C before analysis.

### Blood biomarker measurements

*Single Molecule Array (Simoa)*: The Simoa assays were performed using the Simoa™ NF-light Advantage Kit (item 103,400), Simoa™ mouse Tau Discovery Kit (item 102,209) and run on the Simoa SR-X platform following the manufacturer instruction (Quanterix Corp, Boston, MA, USA) [21].

*AlphaLISA™*: The AlphaLISA assay was performed using the Mouse Matrix Metalloproteinase 9 (MMP9) kit (item AL519C, Revvity) for MMP9. AlphaLISA signals were measured using an Ensight Multimode Plate Reader (Revvity) [21].

### Lectin pathway activity assay

The LP activity was measured in mouse plasma samples obtained at 4 days after TBI or sham injury. For ex vivo LP assessment, EDTA-plasma samples (2.5% final plasma concentration) were incubated on mannan-coated ELISA plates for 15 min to initiate activation followed by detection of C3c deposition as described elsewhere [22]. Briefly, EDTA-plasma samples were thawed on ice and suspended in barbital buffered saline (BBS; 4 mM barbital, 145 mMNaCl, 2 mM CaCl_2_, 1mM MgCl_2_, pH 7.4), to a final plasma concentration of 2.5%. Plasma solutions were incubated on the coated plate at 37 °C for 15 min. The plate was washed and incubated for 1 h 30 min at RT with a polyclonal anti-C3c antibody (Dako, A0062) diluted 1:5000 in washing buffer. After washing, the plate was incubated with an alkaline-phosphatase labelled goat anti-rabbit IgG antibody (Sigma A-3812) diluted 1:5000 in washing buffer for 1 h 30 min at RT. Following washing, the assay was developed by adding 100 µL substrate solution (Sigma Fast p-Nitrophenyl Phosphate tablets, Sigma) and the absorption at 405 nm measured using the Infinite M200 spectrofluorimeter managed by Magellan software (Tecan, CH).

### Histological analysis

#### Sacrifice and tissue collection

Mice were transcardially perfused with chilled paraformaldehyde (4% in PBS). The brains were transferred to 30% sucrose in PBS at 4 °C overnight for cryoprotection. Then they were frozen by immersion in isopentane at − 45 °C for 3 min before being sealed into vials and stored at -80 °C until use. For lesion size determination, twenty-micron coronal brain cryosections were cut serially at 200-µm intervals and stained with cresyl violet (Sigma Aldrich).

#### Assessment of contusion volume

Eight coronal sections from bregma + 1.2 mm to − 4 mm were acquired from each mouse and visualized at 2x magnification with an Olympus BX-61 Virtual Stage microscope with a pixel size of 3.49 mm. The injured area was calculated by subtracting the contralateral hemisphere minus the ipsilateral hemisphere as previously described [13].

#### Neuronal loss

Three 20-µm coronal sections at 0.4, 1.6, and 2.8 mm posterior to bregma and stained with Cresyl violet (Sigma-Aldrich, St. Louis, MO) were selected from each mouse brain to quantify neuronal cell loss. The entire sections were acquired with an Olympus BX-61 Virtual Stage microscope using a 20x objective lens, with a pixel size of 0.346 μm. Acquisition was done over 10-µm thick stacks with a step size of 2 μm. The different focal planes were merged into a single stack by mean intensity projection to ensure consistent focus throughout the sample. Neuronal count was performed by segmenting the cells over a cortical region located within 350 μm from the contusion edge and in the corresponding contralateral hemisphere. The segmented cells with a round-shaped signal sized < 25 mm^2^, corresponding to glial cells, were excluded from the analysis. Quantification was performed by Fiji software. Neuronal loss was calculated as follow: 1- (#neurons in ipsilateral cortex / #neurons in contralateral cortex). Value = 0 indicates no neuronal loss.

### Study design and randomization

The study was designed in two parts, a pilot study to compare α-MASP-2 to the control Ab and a follow-up study to compare α-MASP-2 to α-MASP-1 and control Ab. In both studies we produced a randomization list using *randomize.org*. Mice were randomly allocated to surgery (sham or TBI) and to the treatment in a balanced manner through the different experimental days. For the pilot study, we dedicated two days for surgeries, each with a total of 24 operated mice. The allocation ratio in each day was 1:1 for surgery (sham: TBI) and treatment (α-MASP-2:control Ab). For the follow-up study, we dedicated four days for surgery, three with a total of 20 and one with a total of 18 operated mice. The allocation ratio in each day was 1:3 for surgery (sham: TBI) and 1:1:1 for treatment (α-MASP-2: α-MASP-1:control Ab). A detailed report of mice allocation to experimental days and groups of the follow-up study is accessible in the online data repository linked to this paper (see ‘Availability of data and materials’).

### Sample size and statistics

The first part of the study, in its explorative nature, relied on the usual numerosity applied for this type of study, i.e. a *n* = 12 per experimental group and for both sham and TBI mice. When we designed the comparative study between α-MASP-2 and α-MASP-1, we pre-defined a primary study endpoint – the decrease in cognitive deficits calculated by Barnes maze test – which was used to calculate the sample size. We considered a 50% cognitive deficit reduction, corresponding to actual animals’ better performance (e.g., half of the time spent to reach the escape tunnel compared to the expected time of 40 s for a TBI mouse). Group size was 19 defined by the formula: *n* = 2σ^2^f(α,β)/Δ^2^ (SD in groups = σ, type 1 error α = 0.02, type II error β = 0.2, the percentage difference between groups Δ = 50). The standard deviation used in the formula for each assessment was calculated based on previous experiments with the same outcome measure (e.g., latency seconds to reach the escape tunnel), resulting in σ = 49 and *n* = 19. To avoid an excessive total number of mice and being mainly interested in the comparisons among TBI mice, we decided to limit the number of sham mice at *n* = 7. A further reason of doing so was that the pilot study indicated a limited variability in the sham group assessing the latency time to escape in the Barnes maze. The data distribution was assessed using tests for normality (D’Agostino & Pearson, Shapiro-Wilk, or Kolmogorov-Smirnov tests). Group comparisons were conducted using t-tests or Mann-Whitney tests, or relevant two-way analysis of variance (ANOVA) followed by the appropriate post hoc test. Equal variances were checked by Bartlett’s test and, if not equal, a Welch’s correction was applied to the test. The identification of outliers was performed by ROUT method setting Q to 0.5%. Any removed outlier is detailed in the figure legends. Statistical analyses were performed using standard software package GraphPad Prism (GraphPad Software Inc., San Diego, CA, USA, version 9.0). All data were presented as mean and standard deviation (SD). *P*-values lower than 0.05 were considered statistically significant.

## Results

**Fig. 1:**
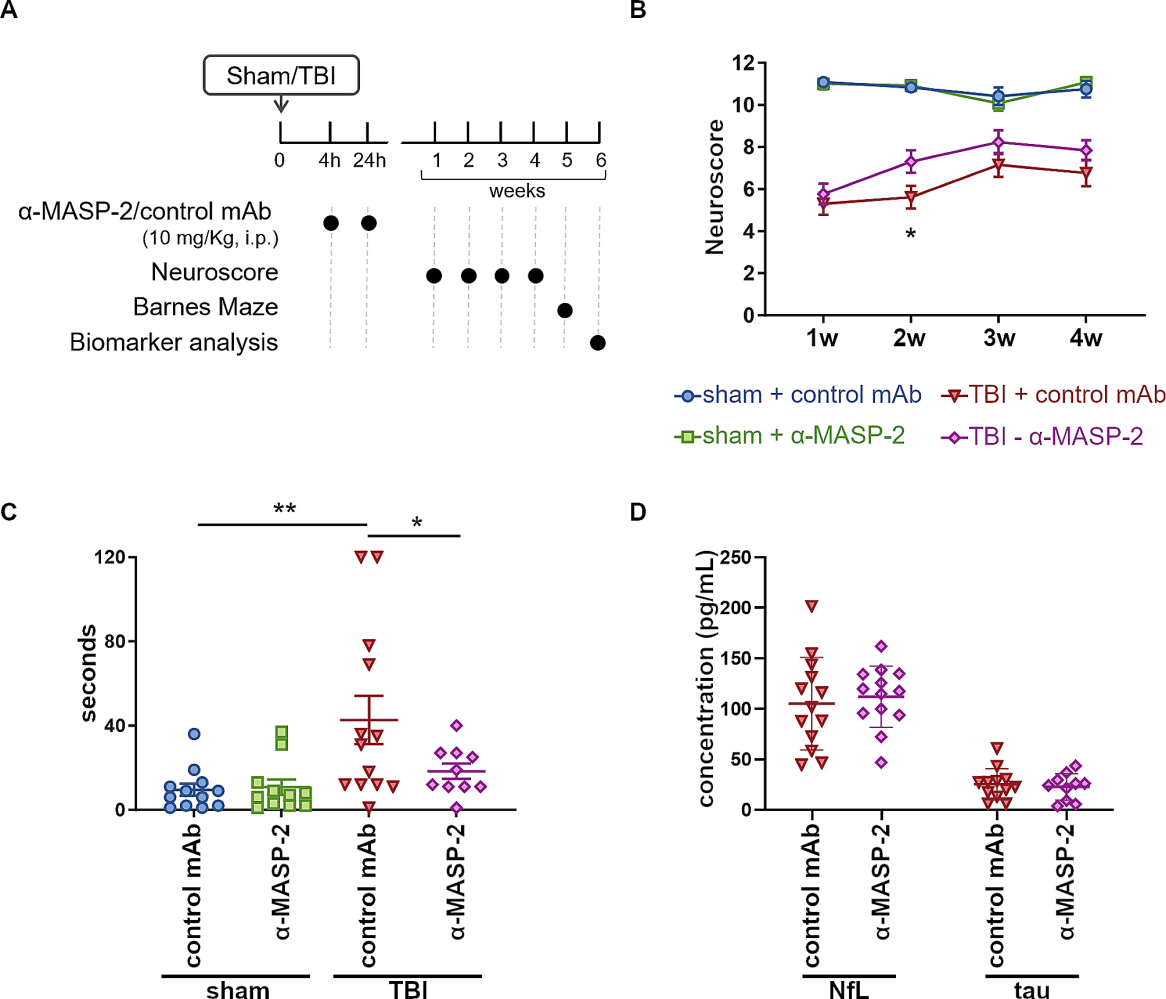
MASP-2 inhibition protects from sensorimotor and cognitive deficits after TBI. (**A**) The experimental plan. (**B**) Sensorimotor deficits were assessed using neuroscore from 1 to 4 weeks following TBI or sham surgery. The neuroscore showed slight, but significant better recovery in the α-MASP-2 treated TBI mice. Two-way ANOVA for repeated measures (p of surgery < 0.0001) followed by Tukey’s post-hoc comparison test, *p < 0.05. (**C**) Cognitive deficits were assessed by Barnes maze test. The α-MASP-2 treated mice showed significantly reduced latency to false escape in the probe trial compared to control mAb treated mice. Two-way ANOVA, p of surgery: <0.01. Tukey’s post-hoc test, *p-value < 0.05, **p-value < 0.01. (**D**) Quantification of NfL and tau levels in mouse plasma obtained 6 weeks after TBI. No differences were observed among the treatments in sham or TBI mice. Data in C and D are presented as scattered dot plot, line at mean ± SD

Our previous work demonstrated that, compared to other complement targets, ablation of MASP-2 was associated with the highest degree of protection in the mouse CCI model of TBI [13]. To evaluate the therapeutic potential of MASP-2 inhibition using clinically relevant treatment approaches, a pilot study was conducted according to the plan depicted in Fig. 1A. The treatment consisted of α-MASP-2 administration at 4 and 24 h post-TBI and mice were followed-up for 6 weeks.

TBI mice receiving α-MASP-2 experienced faster recovery of sensorimotor function, which was evident 2 weeks after TBI, and a notable amelioration of the cognitive deficits per the Barnes maze test at 5 weeks after TBI (Fig. 1B and C). Importantly, the α-MASP-2 treated TBI mice took 24 s less than control mAb treated TBI mice to reach the escape hole during a 2-minute test, which is a large reduction of the cognitive deficits. The analysis of the circulating biomarkers NfL and tau, performed at 6 weeks after TBI, showed no difference between the groups (Fig. 1D).

**Fig. 2:**
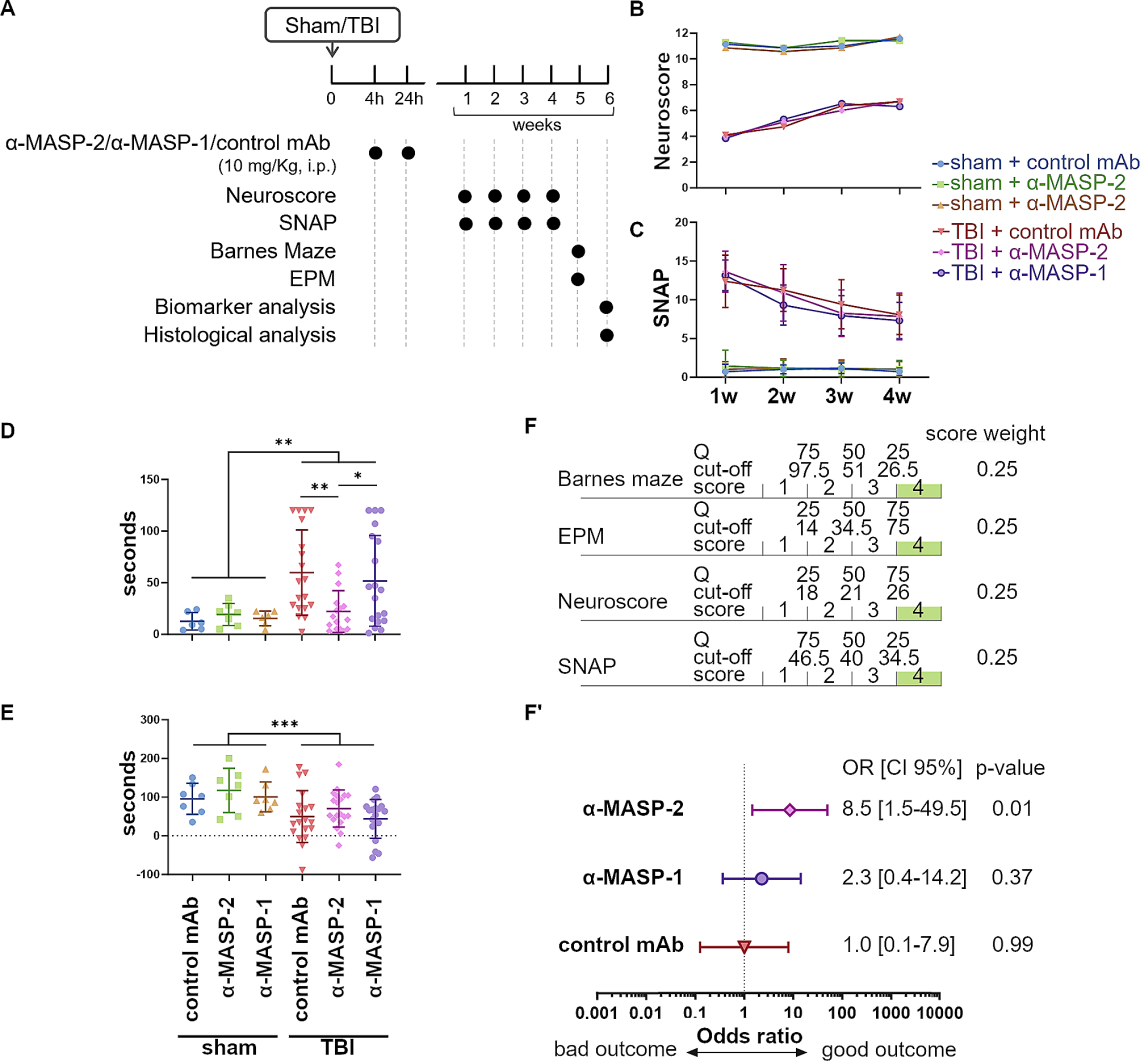
MASP-2, but not MASP-1 inhibition ameliorates the outcome of TBI mice. **A**) The experimental plan. **B**, **C**) Sensorimotor deficits were assessed using neuroscore (**B**) and SNAP (**C**) from 1 to 4 weeks following TBI or sham surgery. Neither the neuroscore nor the SNAP tests showed significant differences between the treatments. Two-way ANOVA for repeated measures (p of surgery < 0.0001) followed by Tukey’s post-hoc test. Data are presented as the mean ± SD. **D**) Cognitive deficits were assessed by Barnes maze test. The α-MASP-2 treated mice showed significantly reduced latency to false escape in the probe trial compared to α-MASP-1 or control mAb treated mice. Two-way ANOVA, p of surgery: <0.01. Tukey’s post-hoc test, *p-value < 0.05, **p-value < 0.01, ***p-value < 0.001. Outlier identification per Rout, Q 0.5%:1 outlier sham control mAb, 1 outlier sham α-MASP-1, 3 outliers TBI α-MASP-2. Data are presented as scattered dot plot, line at mean ± SD. **E**) Anxious behavior was assessed by the elevated plus maze and expressed as seconds spent in the closed arms minus those in the open arm. Two-way ANOVA, p of surgery < 0.001. Data are presented as scattered dot plot, mean ± SD. **F**, F’) Association of the treatments with the functional outcome of TBI mice over 5 weeks. The health score was obtained by rating mice from 4 (good outcome) to 1 (bad outcome) based on quartiles (Q) of the Barnes, EPM, neuroscore and SNAP values over the 5 weeks of observation. The final score was the sum of the weighted scores of the four parameters each accounting for 25% of the final score (**F**). The odds ratio was calculated by a Chi square test using the Woolf logit interval for calculating the 95% confidence interval (CI 95%), stratifying mice in terms of good outcome (defined as a score > 3) versus bad outcome (score ≤ 3). Forest plot showing that the α-MASP-2 treatment was significantly associated with a good outcome (F’)

To confirm the findings of the pilot study, we evaluated the efficacy of α-MASP-2 treatment in a follow-on study using a larger cohort of mice. This study also included an additional group of TBI mice treated with α-MASP-1. This was done because previous reports suggested that MASP-1 may be needed for efficient MASP-2 and LP activation [6], whereas our previous work showed that MASP-1/3-deficient mice (which lack MASP-1 but also MASP-3 required for activation of the alternative pathway of complement) were not protected in the TBI model [13]. By comparing the inhibition of MASP-1 and MASP-2, we aimed to dissect the roles of LP activation enzymes in the context of TBI. We followed the experimental plan depicted in Fig. 2A and prespecified cognitive deficits evaluated by the Barnes maze at 5 weeks after TBI as the primary outcome.

While improving gradually over time, TBI-induced sensorimotor deficits persisted till the 4th week of observation, and neither antibody treatment improved recovery of sensorimotor function compared to isotype control (Fig. 2B and C). Cognitive assessment by the Barnes maze showed increased latency to false escape after TBI in control antibody-treated mice. The treatment with α-MASP-2 significantly improved cognitive deficits compared to isotype control or α-MASP-1 treatment (latency of 22.06 ± 20.28 s compared to 51.74 ± 43.89 s [*p* < 0.01] and 59.68 ± 41.42 [*p* < 0.05], respectively) 5 weeks after TBI (Fig. 2D). In the elevated plus maze, control mAb-treated TBI mice showed higher anxiety as indicated by less time spent in the closed than open arms compared to sham mice. While α-MASP-2 treated mice showed a trend toward improvement, the effect was not statistically significant, and no treatment effect was apparent for α-MASP-1 treatment (Fig. 2E).

To obtain a comparative evaluation across treatment groups, a health score based on the four behavioral tests (neuroscore, SNAP, Barnes, and EPM) was calculated. Mice were rated from 1 (bad outcome) to 4 (good outcome; Fig. 2F) based on quartile distribution calculated for the isotype control-treated mice. The α-MASP-2 treatment was the most protective, showing a positive association with a good outcome (odds ratio 8.5 [95% CI 1.5–49.5], *p* = 0.01). The α-MASP-1 treatment showed a weaker, non-significant association with a good outcome (odds ratio 2.3 [95% CI 0.4–14.2], *p* = 0.37, Fig. 2F).

**Fig. 3:**
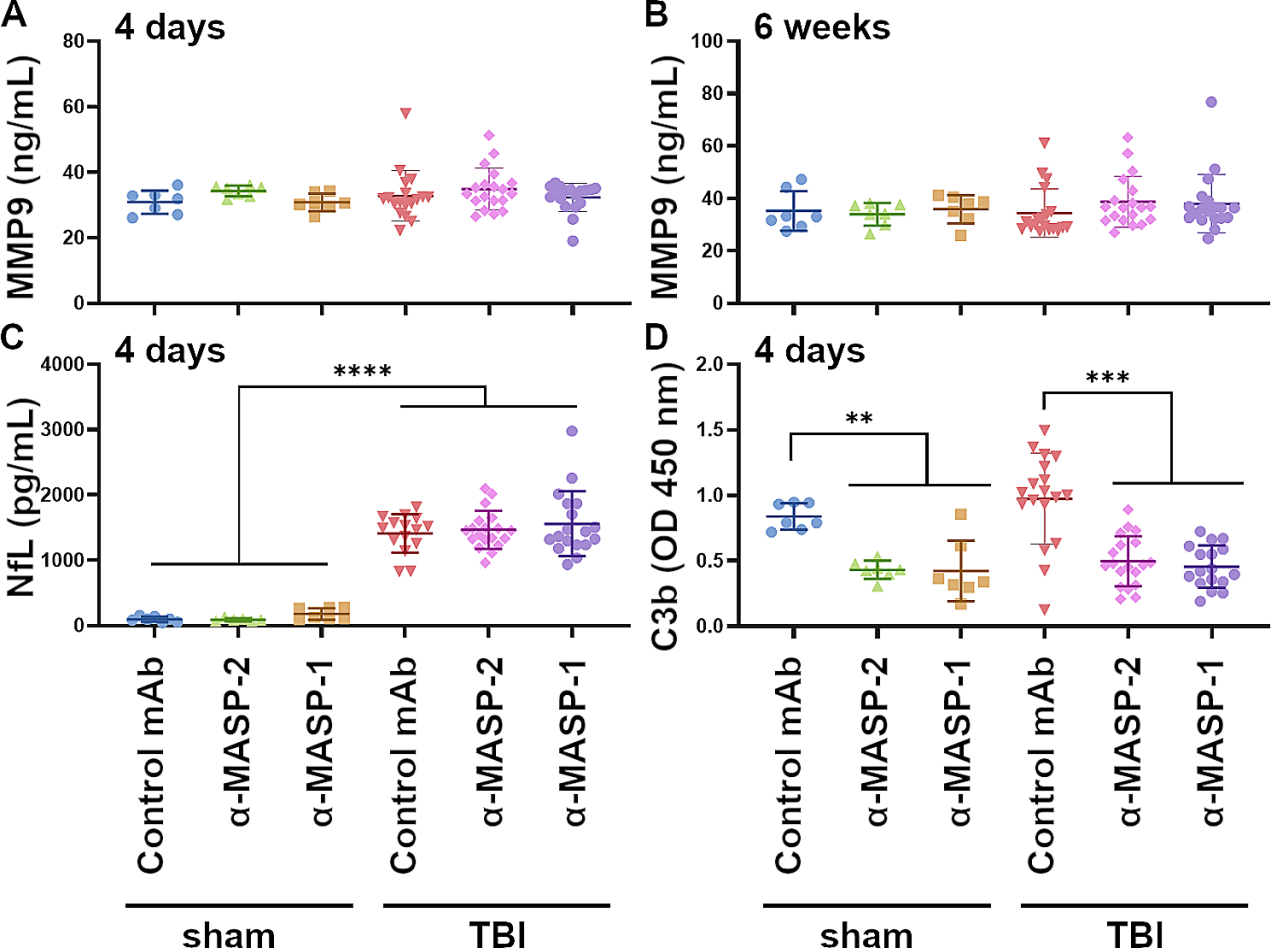
MASP-2 or MASP-1 inhibition reduce LP activation, but not the levels of circulating biomarkers of neuronal or vascular damage. **A**) Quantification of MMP9 levels in mouse plasma obtained 4 days (**A**) and 6 weeks (**B**) after TBI or sham injury. No differences were observed among the treatments in sham or TBI mice. **C**) Measurement of NfL levels in mouse plasma obtained 4 days after TBI or sham injury. NfL levels increased significantly after TBI without any difference among the treatments. Two-way ANOVA, p of surgery < 0.0001. Data are presented as scattered dot plots, mean ± SD. **D**) Quantification of C3b deposition on mannan-coated plates exposed to mouse plasma collected 4 days after TBI/sham surgery. Mice treated with α-MASP-2 and α-MASP-1 showed a significant reduction in C3b levels, indicating a reduced activation of the lectin pathway. Two-way ANOVA, Tukey’s post-hoc test, **p-value < 0.01, *** *p*-value < 0.001. Data are presented as scattered dot plot, line at mean ± SD

MMP9 levels did not differ between sham and TBI mice at 4 days or 6 weeks after TBI, nor were the levels affected by treatment (Fig. 3A, B). As for NfL, while TBI mice showed higher plasma levels compared to sham animals 4 days after TBI as expected, no treatment effects were detected (sham: control Ab: 98.5 ± 41.24 pg/ml ± SD; α-MASP-2: 90.51 ± 22.89; α-MASP-1: 180.0 ± 87.36; TBI: control Ab: 1411 ± 293.6; α-MASP-2: 1467 ± 290.3; α-MASP-1: 1559 ± 494.8, Fig. 3C).

To verify LP suppression in α-MASP-1 and α-MASP-2-treated mice, we evaluated LP functional activity in plasma samples collected during the treatment phase using ex vivo functional assays [15, 23]. As expected, both α-MASP-1 and α-MASP-2 treatment resulted in a significant reduction of LP-induced C3b deposition compared to isotype control treatment in both sham and TBI mice (Fig. 3D).

**Fig. 4:**
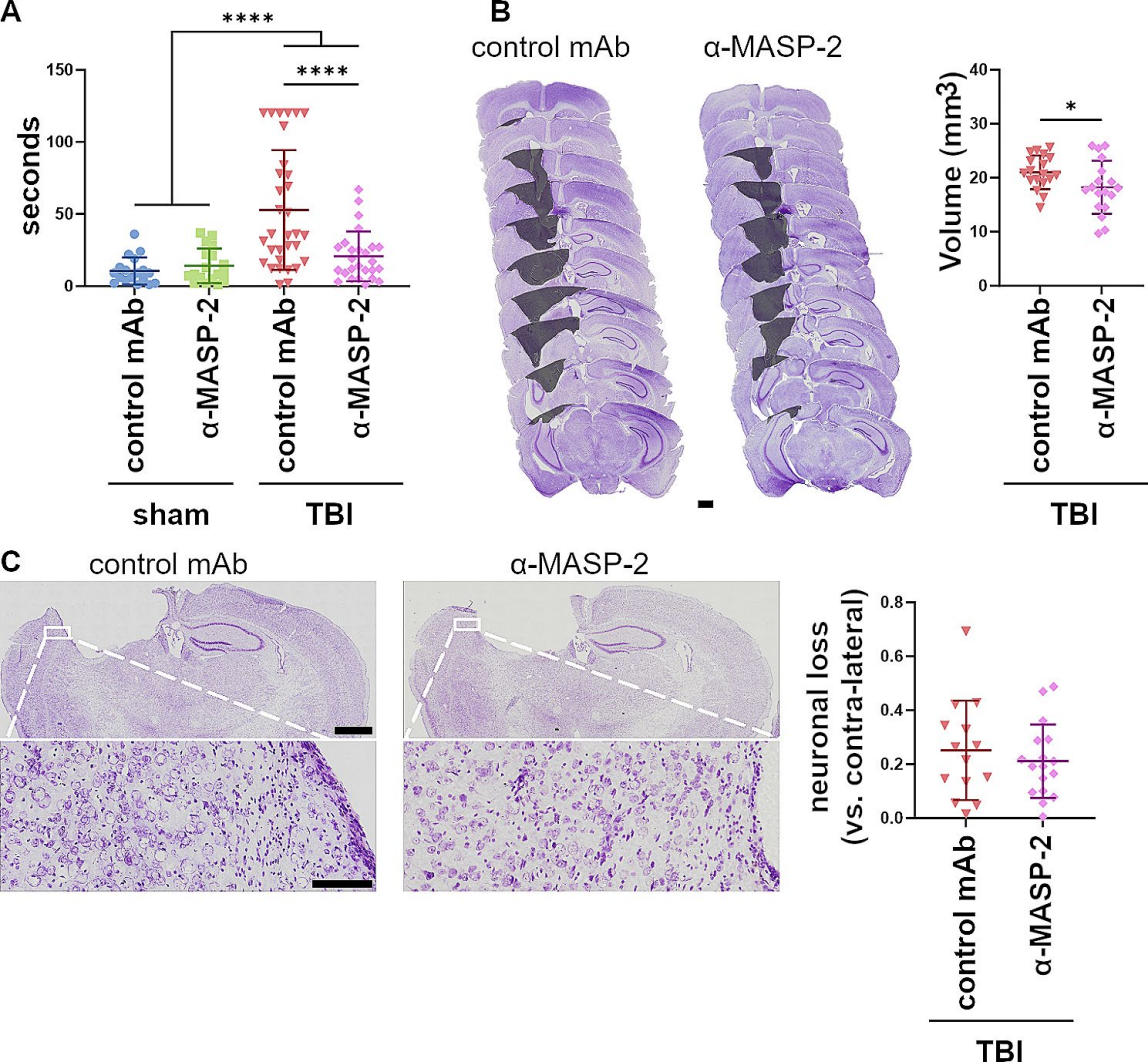
Aggregated data from the two studies relative to the α-MASP-2 treatment. (**A**) Barnes maze test showing a strong amelioration of cognitive deficits in the α-MASP-2 treated TBI mice at 5 weeks after injury. Two-way ANOVA, p of surgery: <0.0001. Tukey’s post-hoc test, **** *p*-value < 0.0001. (**B**) Lesion volume. The microphotographs (scale bar 1 mm) indicate the lesion induced by the CCI model (colored surface). Visual inspection and volume quantification showed a decreased size of the lesion in the α-MASP-2 treated TBI mice at 6 weeks after injury. Unpaired t-test, * *p*-value < 0.05. (**C**) Neuronal loss. The microphotographs (scale bar 1 mm) show brain slices at the maximal burden of the lesion, with inserts (scale bar 100 μm) highlighting the Cresyl violet staining in the proximity of the contusion edge, where the quantification of viable neurons was done. The neuronal loss did not differ between groups at 6 weeks after injury. Unpaired t-test, *p*-value = 0.49. All data are presented as scattered dot plot, line at mean ± SD

In our final analysis, we gathered the data from all mice treated with α-MASP-2 in the two studies and included a histological analysis of the lesion. We observed that TBI mice treated with the α-MASP-2 had a cognitive improvement (52.78 ± 41.51 vs. 20.56 ± 17.23 latency seconds ± SD; Cohen’s d = 1.01, Fig. 4A), which was associated with a decreased lesion volume (18.24 ± 4.91 versus 21.03 ± 3.10; Cohen’s d = 0.68, Fig. 4B) compared to mice receiving isotype control treatment. In contrast, no differences in neuronal loss were observed (Fig. 4C).

## Discussion

In this series of studies, we aimed to evaluate an interventional treatment schedule targeting MASP-2, the effector enzyme of the LP, whose detrimental roles in TBI were previously documented in experimental TBI models [13]. The data reported herein show that treating TBI mice with α-MASP-2 for a short time post-TBI (α-MASP-2 administered at 4- and 24-hours post-TBI) reduced the cognitive deficits at 5 weeks post-TBI.

A key role for MASP-2 in TBI pathophysiology was previously suggested by Osthoff et al. based on an observational clinical study reporting that high circulating MASP-2 levels early after injury were associated with poor outcomes at 90 days post-TBI [12]. These data were paralleled by our own observations in patients with severe TBI, which documented increased intracerebral levels of MASP-2 at the contusion site compared to controls [8]. Elevated MASP-2 brain levels were associated with increased TBI severity, as identified by altered pupillary reactivity and the presence of subarachnoid hemorrhage [8]. Collectively, these data support the evaluation of α-MASP-2 as a potential treatment to counteract and mitigate brain damage and improve outcomes after TBI.

The α-MASP-2 used in this study is a derivative of narsoplimab, an experimental drug that has already been evaluated in clinical trials for stem cell transplant-associated thrombotic microangiopathy, COVID-19 infection, and glomerular diseases. As such, the molecule has already shown clinical safety and effective targeting of MASP-2 in humans, holding the promise for its future use in TBI patients. Young adults represent the largest demographic among severe TBI patients, with men facing more than double the risk compared to women [24] thus we first tested our intervention in male mice. However it is important to note that women tend to experience poorer outcomes, particularly in terms of post-injury depression and anxiety [25], highlighting the importance to investigate sex-dependent pathological mechanisms. Furthermore, TBI epidemiology is shifting to the old-age individuals, where sex differences in TBI prevalence are less prominent and comorbidities may play a major role in modifying disease trajectories. Therefore, future studies should be conducted to extend our research to experimental models incorporating biological variables such as sex, age, and comorbidities to accurately assess the efficacy of α-MASP-2.

The treatment schedule in the present study was designed according to a time-to-treatment which is feasible in the clinical setting, i.e. the first α-MASP-2 dosing was administered 4 h after the injury, and tested in two independent cohorts of mice. The second cohort also included a group treated with α-MASP-1 for comparison and prespecified improvement in the long-term cognitive performances (Barnes maze test 5 weeks after TBI) as the primary endpoint. In aggregate, the study demonstrated that inhibition of MASP-2, but not of MASP-1, improved the cognitive deficits in the TBI mice with a modest protection of the sensorimotor functions. We also observed smaller lesion volumes at sacrifice (6 weeks after TBI) with α-MASP-2 treatment, but no preservation of the numbers of neurons at the contusion edge. This latter observation aligns with the observation that circulating NfL levels were not affected by treatment.

A previous report showed that complement activation via LP promotes blood-brain barrier breakdown and exacerbates axonal damage after TBI [26]. We can hypothesize that at least part of the beneficial effects associated with MASP-2 inhibition may be due to reduction of the vasogenic edema, which was mirrored by the reduced volume of the lesion. To explore this further, we analyzed the blood biomarker of vascular injury MMP9 as early as 4 days after TBI. However, MMP9 levels were unchanged after TBI thus limiting its utility as a proxy for vascular damage in our study. By contrast, NfL blood levels were increased in TBI vs. sham mice. Given the role of the LP in exacerbating the axonal damage, it is unclear why we were unable to observe a reduction of circulating NfL. This observation suggests that a further optimization of the treatment schedule may be needed for optimal efficacy of α-MASP-2 treatment.

Of note, while both α-MASP-2 and α-MASP-1 treatment suppressed systemic LP activity as demonstrated by reduced ex vivo LP activity in plasma samples collected 4 days after sham or TBI surgery, only the inhibition of MASP-2 resulted in appreciable protection from TBI. While we have no clear explanation for the apparent lack of protection for α-MASP-1 treatment, the results presented here are consistent with our previous work comparing experimental TBI outcomes in MASP-2-deficient and MASP-1/3-deficient mice, suggesting that commonly used LP activity assays, such as the ex vivo assay used in this study, are too blunt to assess the relative effectiveness of different LP blocking agents. Briefly, in the assay we incubated plasma on mannan-coated plates and measured the formation of C3b active fragments, which requires C4bC2 convertases downstream to recognition of danger signals by MBL. MASP-2 was reported to induce direct cleavage of C3 into its active fragments in absence of C2 and C4 in specific circumstances, including ischemia/reperfusion injury of the gut or the brain [27]. If our in vitro LP activation assay failed to reproduce this direct mechanism of C3 cleavage by MASP-2, which might occur in vivo, it is explained why, although the inhibition of both MASPs counteracted the LP activation in vitro, only that of MASP-2 was protective after TBI.

A different interpretation of our results relies on the observation by Degn et al., who have reported MASP-2 activation downstream to a rapid MASP-1 activation [28]. While the hypothesis is not fully supported by our previous observations in MASP-1/3-deficient TBI mice, if this is the case for our TBI model, the first intervention at 4 h might have been too late for inhibiting MASP-1, but well timed for MASP-2, which was already activated at the point of intervention. Thus, only MASP-2 inhibition would be effective, supporting the therapeutic approach proposed by our study. As we analyzed the LP activation at 4 days after TBI, it is possible that we selected a too late time-point to display relevance for the acute injury, when activated MASPs are relatively quickly inhibited by circulating enzyme inhibitors such as C1inh and alpha-2-macroglobulin. Hence the LP activation at day 4 could rely on renewed MASP activation, which was efficiently inhibited by α-MASP-1 or α-MASP-2.

## Conclusions

Our data point to inhibition of the essential LP effector enzyme MASP-2 as a promising therapeutic intervention for the treatment of TBI. Among the key aspects of our study are: (1) we applied a clinically relevant protocol with a post-TBI treatment, (2) the study was done in two independent cohorts, demonstrating a similar amelioration of cognitive deficits, (3) we used an inhibitory antibody derived from an antibody that has already completed multiple late-stage clinical trials in other indications, and (4) we targeted a hepatically expressed plasma protein, thereby assuring target blockade in the brain without the antibody needing to cross the blood-brain barrier.

## Data Availability

All data will be publicly available at this online repository on Figshare: 10.6084/m9.figshare.25508812.

## Abbreviations

CCI: Controlled cortical impact
CI: Confidence interval
EPM: Elevated plus maze
i.p.: Intraperitoneal
LP: Lectin pathway
MASPs: Mannan binding lectin associated serine proteases
MBL: Mannan binding lectin
MMP9: Matrix matalloproteinase-9
NfL: Neurofilament light chain
SD: Standard deviation
SNAP: Simple Neuroassessment of Asymmetric imPairment
TBI: Traumatic brain injury
